# Striatal Control of Movement: A Role for New Neuronal (Sub-) Populations?

**DOI:** 10.3389/fnhum.2021.697284

**Published:** 2021-07-20

**Authors:** Tim Fieblinger

**Affiliations:** Institute for Synaptic Physiology, University Medical Center Hamburg-Eppendorf, Hamburg, Germany

**Keywords:** striatum, spiny projection neuron, Parkinson’s disease, single-cell RNA sequencing, scRNAseq, movement, L-DOPA-induced dyskinesia

## Abstract

The striatum is a very heterogenous brain area, composed of different domains and compartments, albeit lacking visible anatomical demarcations. Two populations of striatal spiny projection neurons (SPNs) build the so-called direct and indirect pathway of the basal ganglia, whose coordinated activity is essential to control locomotion. Dysfunction of striatal SPNs is part of many movement disorders, such as Parkinson’s disease (PD) and L-DOPA-induced dyskinesia. In this mini review article, I will highlight recent studies utilizing single-cell RNA sequencing to investigate the transcriptional profiles of striatal neurons. These studies discover that SPNs carry a transcriptional signature, indicating both their anatomical location and compartmental identity. Furthermore, the transcriptional profiles reveal the existence of additional distinct neuronal populations and previously unknown SPN sub-populations. In a parallel development, studies in rodent models of PD and L-DOPA-induced dyskinesia (LID) report that direct pathway SPNs do not react uniformly to L-DOPA therapy, and that only a subset of these neurons is underlying the development of abnormal movements. Together, these studies demonstrate a new level of cellular complexity for striatal (dys-) function and locomotor control.

## Introduction

The striatum is an evolutionarily conserved brain area and input structure to the basal ganglia (Grillner et al., 2013). Functionally, it is a critical hub for the control of locomotion. The classical “box-and-arrow” model of the basal ganglia postulates that the *direct* and *indirect* pathways, originating in the striatum, work in antagonistic ways to exert locomotor control (Albin et al., 1989; DeLong, 1990). Lesion or loss-of-function of the *direct* pathway reduces locomotion in animals, whereas disabling the *indirect* pathway results in hyperlocomotion (Durieux et al., 2009, 2012; Bateup et al., 2010). This is furthermore corroborated by optogenetic studies, showing that overt activation of the *direct* pathway induces locomotion, and activation of the *indirect* pathway leads to a cessation of ongoing movement (Kravitz et al., 2010). The “box-and-arrow” model has been instrumental to a better understanding of the network changes underlying movement disorders and locomotor dysfunction; however, it has become apparent that the model does not reflect the true complexity of the basal ganglia network (Calabresi et al., 2014; Plotkin and Goldberg, 2019). For example, *in vivo* imaging of striatal neurons in freely moving animals has shown that both pathways are simultaneously active during self-initiation of movements (Cui et al., 2013) and it is the coordinated and clustered activity of both pathways that is lost in a mouse model of Parkinson’s disease (PD; Parker et al., 2018). Nevertheless, it is undisputed that the striatum is a key region for locomotor control, and that SPN dysfunction leads to severe motor deficits.

PD patients suffer from loss of normal motor function, caused by the degeneration of dopamine (DA) producing neurons in the substantia nigra. The subsequent lack of DA signal in the striatum causes the hypo- and bradykinetic symptoms (Schneider and Obeso, 2015). Post mortem studies showed that striatal neurons do not *per se* degenerate in PD, yet they become atrophic with loss of dendrites and dendritic spines (McNeill et al., 1988; Stephens et al., 2005; Zaja-Milatovic et al., 2005). This has also been observed in rodent models of PD (Fieblinger and Cenci, 2015). Treatment with L-DOPA is the current gold-standard therapy to restore motor function in PD patients, with typically good responses for bradykinesia and rigidity, yet lesser efficacy for other symptoms, such as posture and gait problems or tremor (for a recent review of PD treatment, see Lee and Yankee, 2021). However, L-DOPA’s benefits come at a price. The majority of patients experience involuntary movements, L-DOPA-induced dyskinesia (LID), with prolonged treatment (Ahlskog and Muenter, 2001). Animal research has, over decades, advanced our understanding of the mechanisms underlying the occurrence of LID. Yet, an effective treatment is still missing. One hurdle has always been the complex and vastly heterogenous organization of the striatum.

In this mini review article, I will shortly recapitulate the anatomical, compartmental, and neuronal divisions of the striatum, which create a complex and overlapping field of diversity. Recent studies using single-cell transcriptomics have now shed new light on this issue. The transcriptional profiles of striatal neurons harbor a code for their anatomical and compartmental identity, and also reveal the existence of previously unknown, discrete neuron populations and sub-populations. How these newly identified neurons participate in the overall striatal function and locomotor control is however yet to be determined. In a parallel development, studies investigating striatal neurons in animal models of LID have shown that—in contrast to previous expectations—not all striatal neurons of a given class are equally contributing to the generation of abnormal movements. Together, these developments suggest a previously overlooked genetic diversity of striatal neurons that might be critically linked to locomotor control and neurological disorders affecting the striatum.

## Divisions of The Striatum

**Figure 1:**
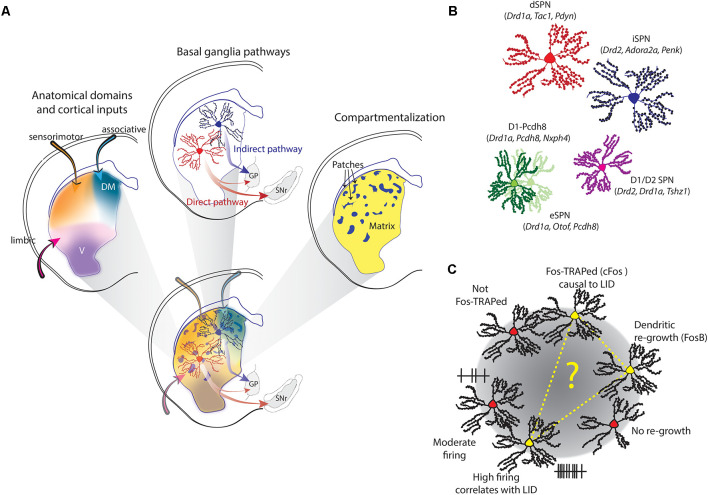
Striatal heterogeneity, spiny projection neuron (SPN) (sub-) populations and L-DOPA-induced dyskinesia (LID)-associated dSPNs. **(A)** The rodent striatum constitutes a complex, overlapping field of heterogeneity. It combines anatomic-functional domains, patch/matrix compartmentalization, and the “classical” distinction of dSPNs and iSPNs, which anchor the *direct* and *indirect* pathway of the basal ganglia. **(B)** Apart from “classical” SPNs, also D1/D2-SPNs were described in scRNA-seq studies, as well as new sub-populations. Characteristic genes are given in parentheses. **(C)** Three different studies identified a sub-group of dSPNs specifically linked to LID. The sub-group was either identified by Fos-TRAP, high-firing activity that correlates with LID, or a specific cellular phenotype. It is tempting to speculate that these three markers identified the same LID-linked subgroup of dSPNs.

The striatum is one of the largest structures in the rodent brain. Although lacking clear internal demarcations, the striatum has a complex organization which divides it along several, overlapping parameters (Figure 1A).

## Striatal Anatomical Domains and Cortical Input Zones

The striatum is commonly divided into a dorsolateral (DL), -medial (DM), and ventral domain (Figure 1A). Each domain is not marked by anatomical borders but defined through functional differences (Yin and Knowlton, 2006; Graybiel and Grafton, 2015). While the DL striatum is dominantly involved in sensorimotor function (e.g., locomotor control and habit formation), the DM striatum takes preferentially part in associative tasks (e.g., goal-directed behavior) and the ventral striatum is often considered part of the limbic system and thus involved in e.g., motivational behavior. This function-based anatomical division is also reflected in the type of inputs these different domains receive. A study mapping different cortical inputs to the striatum showed that they are largely overlapping with the three different domains. There is a distinct DM region of the striatum with highly convergent cortical innervation, a DL region receiving dense sensory-motor inputs, and a ventral region, mostly innervated by limbic areas (Hunnicutt et al., 2016). Based on this type of mapping, the posterior striatum constitutes an additional, fourth anatomical domain. This is in line with previous observations that the posterior striatum receives distinct DA inputs (Menegas et al., 2015) and bears a unique neuronal composition (Gangarossa et al., 2013). The importance of this fourth domain has been recently discussed elsewhere (Valjent and Gangarossa, 2020).

## Striatal Compartments

The second division of the striatum is the distinction of patches (or striosomes) and matrix (Figure 1A). The patches are like islands, constituting around 10–15% of the striatal volume. There are no anatomical demarcations separating the patches, but their existence can be revealed by histochemical markers, such as the μ-opioid receptor or acetylcholinesterase (Herkenham and Pert, 1981). Patch and matrix are associated with different behavioral functions. In rodents, for example, patches are involved in cost-benefit trade-off decisions (Friedman et al., 2015), and in humans, striatal patches play a certain role in cognitive control (Beste et al., 2018). Also, the neurons inside the two compartments differ, for example in their synaptic connectivity and neuromodulation (for a recent review, see Prager and Plotkin, 2019). Importantly, the patch and matrix compartmentalization can be found in all anatomical domains of the striatum.

## Spiny Projection Neuron Populations

The striatum hosts one type of projection neuron: the GABAergic spiny projection neuron (SPN), sometimes referred to as medium-spiny neuron (MSN). The SPNs form the backbone of the basal ganglia and are “classically” divided into two groups, forming the *direct* (dSPNs) and *indirect* pathway (iSPNs). The dSPNs project predominantly to the substantia nigra reticulata (SNr) and express the DA D1 receptor, whereas the iSPNS project to the globus pallidus pars externa (GPe) and express the D2 receptor. They also differ in a range of electrophysiological, morphological, and molecular parameters (Gertler et al., 2008; Heiman et al., 2008; Planert et al., 2013). Importantly, dSPNs and iSPNs are intermingled in the striatum. They are found in all anatomical domains, and both patches and matrix (Figure 1A). However, with regard to locomotor control, the *direct* and *indirect* pathways are classically associated with opposite functions. While the direct pathway is considered to promote motor behavior and initiation of locomotion, the indirect pathway has been associated with the opposite, i.e., inhibition of movement initiation and cessation of ongoing locomotion (Albin et al., 1989; DeLong, 1990; Kravitz et al., 2010).

Some distinctions between the “classical” SPN groups are however not simply “black-or-white” (but rather graded) and controversies prevail (see Calabresi et al., 2014). One point of debate is also a group of SPNs expressing both the D1 and D2 receptors. These constitute only a small fraction of all SPNs (about 2%) but show distinctive electrophysiological and morphological properties (Gagnon et al., 2017). If these D1/D2-SPNs project to the SNr and/or GPe is not yet established. It is also known that dSPN axons do not exclusively terminate in the SNr, but also make very plastic connections in the GPe (Cazorla et al., 2012; Cui et al., 2021). Another open question is whether patches are equally composed dSPNs and iSPNs, or preferentially enriched with one type (see Prager and Plotkin, 2019). Along this line, it has been noted that the most caudal part of the striatum (corresponding to the fourth anatomical division mentioned above) is largely devoid of iSPNs (Gangarossa et al., 2013). New experimental approaches, for example investigating single-cell information, may be useful for answering, at least in part, these open questions.

## Transcriptional Profiles Reveal a Code for Striatal Heterogeneity and New Types of SPNs

Before the advent of single-cell sequencing technology, transcriptional analysis was at large limited to bulk measures from whole tissue or fluorescently labeled and sorted neuronal populations. Single-cell mRNA sequencing (scRNA-seq) provided a major advancement: it enabled the unbiased analysis of a single cell’s transcriptome, for thousands of cells at a time, and thus revealed the individual and populational heterogeneity of SPNs.

One of the first scRNA-seq studies unearthed a range of interesting insights (Gokce et al., 2016). First, it confirmed that SPNs can be classified into dSPNs and iSPNs based on specific and largely known markers. The gene set to identify dSPNs includes, for example, *Drd1a* (the gene coding for the D1 receptor), *Tac1* and *Isl1*, whereas the set to identify iSPNs includes *Drd2* (coding for the D2 receptor), *Adora2a*, *Penk*, *Gpr6*, *Gpr52*, and *SP9*. These sets enabled a discrete and robust separation of the “classical” SPNs, and similar ones were used in subsequent studies (Ho et al., 2018; Saunders et al., 2018; Martin et al., 2019; Malaiya et al., 2020; Stanley et al., 2020). Secondly, it also reported a small group of D1/D2 SPNs. Lastly, it revealed that SPNs within a given class have highly heterogeneous transcriptional profiles. This heterogeneity was used to characterize further subpopulations. For dSPNs a major subpopulation (D1-Foxp1) was identified, characterized by the expression of high levels of *Foxp1* and *Camk4*, as well as a minor subpopulation of dSPNs (D1-Pcdh8) characterized by *Pcdh8*, *Tacr1*, and *Adarb2*. The iSPNs similarly divide into subpopulations: a minor group marked by the unique expression of genes including *Htr7* and *Agtr1a*, and a major subpopulation characterized by a lack of *Cacnad2d3* and *Kcnip1* expression. Interestingly, it was observed that other genes form distinct gradients across all SPNs. The meaning of this gradient was, however, only examined in later studies (see below).

Another extensive scRNA-seq study, investigating several brain regions, confirmed the transcriptomic distinction of dSPNs and iSPNs in the striatum, based on more than 60 differentially expressed genes (Saunders et al., 2018). In contrast to the previous study, it additionally provided evidence for a third SPN population, which was neither a subpopulation of dSPNs nor iSPNs. These so-called “eccentric” eSPNs are only few in numbers, but transcriptionally distinct based on over 100 differentially expressed genes. Some of the eSPN markers encompass genes typically used to distinguish iSPNs and dSPNs, such as *Adora2a* and *Drd1*, which likely explains why this population was overlooked so far. *In situ* hybridization (ISH) further showed that eSPNs, dSPNs and iSPNs are intermingled in the striatum. Key marker genes for eSPNs include *Casz1*, *Otof*, *Cacng5*, and *Pcdh8*—of which the last was also a defining factor of the D1-Pcdh8 subpopulation described previously (Gokce et al., 2016), suggesting a possible relationship between these two groups. A later study, investigating the role of *Foxp1* in striatal neuron development, confirmed the transcriptional distinction of dSPNs and iSPNs in early postnatal mice, as well as the existence of D1/D2-SPNs and eSPNs, based on scRNA-seq (Anderson et al., 2020). *Foxp1* appears to be especially important for the segregation of different SPN populations during the development, as its deletion led to an enrichment of eSPN markers in both dSPNs and iSPNs.

Expanding the first report (Gokce et al., 2016), a subsequent study deeper investigation the transcriptional gradients in SPNs by combining scRNA-seq with ISH (Stanley et al., 2020). Apart from dSPNs and iSPNs two further main populations are defined: the SPNs of the Islands of Calleja (IcjSPNs) and the D1-Pcdh8 population. Key markers for IcjSPNs are the expression of *Drd1a* and *Rreb1*, and *Pcdh8* and *Nxph4* mark D1-Pcdh8. Upon closer examination, the study finds nine subgroups of dSPNs and seven subgroups of iSPNs, all of which were best defined by a combination of genetic markers. Several subgroups also showed a preferential anatomical location, as revealed by ISH. Most interestingly, this study reveals that the transcriptional gradients across all SPNs actually codes for the cells’ anatomical position (along the dorsoventral axis), as well as their compartmental identity. Along the dorsoventral axis, the expression-ratio of *Cnr1* to *Crym* is lowest in the ventromedial, and highest in the DL striatum. This gradient has been recently confirmed in both mouse and marmoset striatum (Martin et al., 2019). It furthermore matched, at least to some extent, the cortical input patterns, as cortical regions enriched in *Cnr1* or *Crym* project to strong *Cnr1* or *Crym* expressing striatal regions. The transcriptional gradient thus nicely aligns with the anatomical domains (Hunnicutt et al., 2016).

Compartmental identity, i.e., whether an SPN belongs to the patches or matrix, was found to be coded by several genes (*Kremen1*, *Sema5b*, and *Id4*), forming a gradient orthogonal to the *Cnr1* to *Crym* ratio (Stanley et al., 2020). Overall, this study confirmed again that scRNA-seq can identify discrete neuron populations, which can both be spatially clustered (like icjSPNs) or intermingled (like dSPN and iSPNs). Furthermore, it showed that information about the anatomical location and compartmental identity is on the other hand not discretely coded, but lies on a continuous gradient, for both dimensions.

The transcriptional signatures of patches and matrix identity were recently further dissected (Martin et al., 2019). Characteristic genes for patches are *Oprm1* (coding for the μ-opioid receptor, a well-described immunohistochemical marker) and *Sema5B*, and *Id4* is a matrix-specific gene. But in addition to these, the authors also describe a curious population of SPNs that is enriched in both, *Oprm1* and *Id4*. These are the so-called exopatch SPNs. Exopatch SPNs are placed in the matrix but physiologically resemble SPNs of the patches (Smith et al., 2016). Based on scRNA-seq data it is concluded that most of the patch and exopatch neurons are dSPNs. Among all dSPNs, this study further identified a distinct subpopulation, characterized by *Col11a1*, *Otof*, *Cacng5*, and *Pcdh8*. This transcriptomic profile resembles closely the previously reported eSPNs (Saunders et al., 2018; Anderson et al., 2020) and/or D1-Pcdh8-SPNs (Gokce et al., 2016; Stanley et al., 2020). Adding to the characterization of this particular SPN group, the authors demonstrate that they project to the SNr, arguing for a close relationship with classical, striatonigral-projecting dSPNs (Martin et al., 2019). It is tempting to speculate that this newly identified SPN group therefore could be promoting locomotion as well.

## Advances and Limitations

The scRNA-seq technology presents certain advantages over previous, population-based approaches such as BAC-TRAP, which is based on EGFP-tagging ribosomal subunits to investigate mRNA undergoing translation in specific cell types. This has been successfully used to describe transcriptome differences of striatal dSPNs and iSPNs (Heiman et al., 2008), and their changes in rodent models of disease (Heiman et al., 2014). However, not all genes that were identified as differentially expressed by BAC-TRAP were confirmed using scRNA-seq, with some apparently not being expressed in SPNs at all (Ho et al., 2018). Consulting complementary approaches (such as independent gene expression databases), it was concluded that the BAC-TRAP results are likely false-positives caused by the sampling of interneurons and “contamination” by mRNA originating from cortical axons (Ho et al., 2018). This shows the utility of scRNA-seq in refining and advancing population-based findings.

On the other hand, the successful isolation of single-cells from a complex tissue is a major challenge for scRNA-seq studies. This is particularly true in a structure like the striatum, which receives a lot of short- and long-distance connections from various other brain areas. Even a simple variation of tissue thickness can be a major source of variability in cellular recovery (Ho et al., 2018). Differences in tissue extraction and cell isolation could therefore limit comparisons across different studies. The scRNA-seq studies reviewed here are however largely in line with each other, with a few minor discrepancies. For example, one study described *Otof* as a marker for eSPNs (Saunders et al., 2018), yet, others find that it also labels a class of GABAergic interneurons (Martin et al., 2019) or is co-expressed with *Penk* and *Tac1* in the ventral striatum (Stanley et al., 2020). Similarly, *Chrm4* was found to be a dSPN-specific gene in one (Ho et al., 2018), but not another study (Gokce et al., 2016). This is a particularly interesting point, because functionally, M4 muscarinic receptors (encoded by *Chrm4*) have been shown to selectively play a role in synaptic plasticity of dSPNs, but not iSPNs (Shen et al., 2015). These examples make the point that for the interpretation of scRNA-seq data validation through other experimental approaches is required. Since most of the neuronal (sub) types lack a single unique genetic marker but are rather defined by a combination of genes/markers, it still stands to question if these combinations (and resulting classifications) are functionally meaningful.

Nevertheless, scRNA-seq studies expanded our understanding of the heterogeneity in the striatum on several levels. First of all, they all confirm the clear distinction between dSPN and iSPNs. Secondly, they deciphered a transcriptional gradients coding for both the anatomical location of a given SPNs, as well as its compartmental identity. Additionally, most studies confirmed the existence of transcriptionally defined eSPNs or eSPN-like D1-Pcdh8-neurons (Gokce et al., 2016; Saunders et al., 2018; Anderson et al., 2020; Malaiya et al., 2020; Stanley et al., 2020), D1/D2-SPNs (Gokce et al., 2016; Martin et al., 2019; Anderson et al., 2020; Stanley et al., 2020) and exopatch neurons (Smith et al., 2016; Martin et al., 2019; Figure 1B).

But what is the importance of these newly defined SPN subpopulations? Their cellular physiology, intracellular signaling networks, morphology, and role in striatal behaviors have not been rigorously assessed, yet. However, clues about the importance of a dSPN subgroup in locomotor control have emerged from the study of PD and LID.

## A New Sub-Group of dSPNs Causes Abnormal Movements?

L-DOPA treatment induces dyskinesia in PD patients and animal models. Strong evidence suggests that abnormal DA signaling and alteration of SPNs underlies aberrant movement control. Especially dSPNs seem to play a prominent role, even though iSPNs show dramatic alterations as well (Fieblinger et al., 2014; Suarez et al., 2014) and chemogenetic manipulation of both dSPNs and iSPNs proved necessary to elicit full LID symptoms in parkinonian mice (Alcacer et al., 2017). Interestingly, recent publications have now shown that not all (alleged) dSPNs react the same to L-DOPA in the parkinsonian brain, and furthermore, only some appear to be causally linked to abnormal involuntary movements.

A first study used targeted recombination in active populations (TRAP) to capture SPNs that express cFOS during dyskinetic episodes and found only a discrete subpopulation being TRAPed (Girasole et al., 2018). It largely, but not exclusively, consisted of dSPNs. Reactivation of this group—but not random dSPNs—using optogenetics induced dyskinetic behavior in the absence of L-DOPA. Inhibition of this TRAPed group conversely interrupts ongoing LID. In a follow-up article using *in vivo* single-cell recordings, it was shown that L-DOPA elicited high firing rates in a specific subset of dSPNs, whose firing rates also correlated with the severity of dyskinetic behavior (Ryan et al., 2018). This strongly suggests that not all dSPNs equally contribute to abnormal movements. What could be the reason for this? Using retroviral labeling of striatonigral SPNs in a rat model of PD we made a surprising finding: their response to L-DOPA was not uniform, but divided into two “clusters,” with distinct morphological and electrophysiological characteristics (Fieblinger et al., 2018). While one subpopulation’s morphological appearance resembled a typical dSPN in the parkinsonian striatum—with marked dendritic regression—the other showed signs of dendritic recovery. The latter were furthermore less excitable than their counterparts. It is known that L-DOPA treatment induces FosB in a subset of striatal neurons (Andersson et al., 1999; Pavon et al., 2006) and we observed that roughly half of the retrogradely labeled dSPNs showed FosB immunoreactivity. Interestingly, FosB staining was predominantly found in the dSPN subgroup that showed dendritic regrowth. Since FosB-expression has been causally linked to LIDs (Cao et al., 2010; Beck et al., 2019), it seems a plausible assumption that also this particular subgroup is specifically linked to LIDs. Each of these studies identified a subgroup of dSPNs through: (i) TRAP; (ii) firing activity; and (iii) cellular phenotype after L-DOPA treatment, that is specifically linked to LIDs (Figure 1C). Although lacking experimental evidence, it is tempting to speculate that this could be one and the same SPN group.

## Outlook

The scRNA-seq studies found evidence for distinct SPN groups outside the classical dSPNs and iSPNs, such as D1/D2-SPNs, eSPNs, and D1-Pcdh8-SPNs. They also demonstrate that transcriptomic heterogeneity plays an important role and differences (e.g., SPN sub-populations, anatomical location and compartmental identify) are coded through the combination of genes and along gradients, rather than in discrete steps.

As discussed, a current limitation to these scRNA-seq studies is that it has yet to be established if transcriptionally defined cell populations indeed have different functional properties, specific behavioral importance, or if they are particularly relevant in diseases like PD and LID. Previously, the generation of BAC transgenic mice and the restricted expression of fluorescent proteins or Cre-recombinase driven by a dSPN or iSPN specific promoter had provided an excellent tool to selectively investigate dSPN and iSPN functionality (Gong et al., 2003). Similar transgenic mouse lines targeting the new neuronal (sub-) groups identified by scRNA-seq would be highly useful to determine their function and advance our understanding of the striatal organization. However, since they are best defined not by a single, unique gene but rather by the combination of several genes, the development of such reporter mice will not be trivial. Until then, studies investigating the properties of these newly defined (sub-) groups will likely rely on anatomical allocation and/or *post hoc* identification e.g., using a combination of histological markers.

A noteworthy question is also how the different transcriptional gradients are created in SPNs. One potential way could be the differential use and regulation of transcription factors. Interestingly, it were also transcription factors (namely cFos and FosB) that guided the discovery of the LID-associated dSPN subgroup. It is tempting to speculate that this group could be identical, for example, with the newly identified SNr-projecting D1-Pcdh8-SPNs. However, this has to be established in future studies.

## Author Contributions

TF drafted and edited the manuscript.

## Conflict of Interest

The author declares that the research was conducted in the absence of any commercial or financial relationships that could be construed as a potential conflict of interest.
